# Selenium suppresses glutamate-induced cell death and prevents mitochondrial morphological dynamic alterations in hippocampal HT22 neuronal cells

**DOI:** 10.1186/s12868-017-0337-4

**Published:** 2017-01-19

**Authors:** Yan-Mei Ma, Gordon Ibeanu, Li-Yao Wang, Jian-Zhong Zhang, Yue Chang, Jian-Da Dong, P. Andy Li, Li Jing

**Affiliations:** 1 Department of Pathology, Ningxia Medical University, Ningxia Key Laboratory of Cerebrocranial Diseases, Incubation Base of National Key Laboratory, Yinchuan, Ningxia 750004 People’s Republic of China; 2 Department of Pharmaceutical Sciences, Biomanufacturing Research Institute and Technological Enterprise (BRITE), North Carolina Central University, Durham, NC 27707 USA; 3 Department of Pathology, Shanxi Traditional Chinese Medicine Hospital, Xi’an, Shanxi People’s Republic of China

**Keywords:** Glutamate toxicity, Autophagy, Mitochondrial fission, Selenium

## Abstract

**Background:**

Previous studies have indicated that selenium supplementation may be beneficial in neuroprotection against glutamate-induced cell damage, in which mitochondrial dysfunction is considered a major pathogenic feature. However, the exact mechanisms by which selenium protects against glutamate-provoked mitochondrial perturbation remain ambiguous. In this study glutamate exposed murine hippocampal neuronal HT22 cell was used as a model to investigate the underlying mechanisms of selenium-dependent protection against mitochondria damage.

**Results:**

We find that glutamate-induced cytotoxicity was associated with enhancement of superoxide production, activation of caspase-9 and -3, increases of mitochondrial fission marker and mitochondrial morphological changes. Selenium significantly resolved the glutamate-induced mitochondria structural damage, alleviated oxidative stress, decreased Apaf-1, caspases-9 and -3 contents, and altered the autophagy process as observed by a decline in the ratio of the autophagy markers LC3-I and LC3-II.

**Conclusion:**

These findings suggest that the protection of selenium against glutamate stimulated cell damage of HT22 cells is associated with amelioration of mitochondrial dynamic imbalance.

## Background

l-Glutamate, the most abundant excitatory neurotransmitter in the nervous system is involved in a wide variety of brain functions and plays a key role in the pathogenesis of many neurological disorders. It is a potent neurotoxin capable of neuronal destruction when present in high concentration. Glutamate-evoked excitotoxicity has been implicated in the etiology of many neurodegenerative diseases including Alzheimer’s disease (AD), Parkinson’s disease (PD), and ischemic stroke [1]. Glutamate-induced cell death is mediated in part by overstimulation of the postsynaptic glutamate receptor system [2] and non-receptor mediated oxidative toxicity [3]. Prolonged exposure to high concentrations of extracellular glutamate promotes oxidative toxicity by activation of mechanisms that negatively impact cysteine uptake into cells via the cystine/glutamate antiporter leading to depletion of glutathione (GSH) [3]. Depletion of GSH causes a downregulation of the cystine-dependent antioxidant system leading to excessive accumulation of reactive oxygen species (ROS) accompanied by oxidative stress. Oxidative stress perturbs mechanisms that regulate Ca2 + homeostasis in the mitochondria and activates pathways that lead to collapse of the mitochondrial membrane polarity and opening of the mitochondrial permeability transition pore (MPTP).

Mitochondria are autonomous double membrane-enclosed organelles present in most mammalian cells, and mainly involved in aerobic respiration. They are dynamic organelles that continuously undergo remodeling by fusion and fission in response to the cellular and environmental cues. Mitochondrial fusion and fission play critical roles in the maintenance of mitochondrial function and regulation of bioenergetic state of the cell. Three dynamin-related GTPases, Mitofusins 1 (Mfn1), Mitofusin 2 (Mfn2) and optic atrophy 1 (Opa1) are required for fusion of the mitochondrial outer and inner membranes in mammalian cells [4–6]; while mitochondrial fission is mediated by the dynamin-like protein-1 (Drp1) and the mitochondrial fission 1 protein (Fis1) [7]. Mitochondrial fusion results in enlargement of the mitochondria through merging of two separate units. In addition, mitochondrial fusion presumably regulates electron transport, mitochondrial metabolism and calcium homeostasis [8]. In contrast, mitochondrial fission is essential to establish new mitochondria and to eliminate defective mitochondria by mitochondrial autophagy [9]. Both processes are tightly controlled and uniformly balanced under physiological conditions.

Autophagy is a vital intracellular catabolic process that causes cellular protein and organelle turnover, through sequestering and priming proteins for lysosomal degradation [10]. Autophagy can be stimulated by a variety of stress-inducing conditions including nutrient depletion, reactive oxygen species, and hypoxia [11]. Activation of this pathway may not necessarily lead to cell death [12]. Defects in the autophagy-regulation and signaling have been associated with many human diseases, including neurodegenerative disorders [13]. Three types of autophagy (macroautophagy, microautophagy, and chaperone-mediated autophagy) have been identified of which, microautophagy has been studies extensively [10]. A number of key signaling pathways ties autophagy to stress responses. Activation of these pathways is orchestrated by a sequence of core autophagy-related (ATG) genes that are evolutionarily conserved. One of the proteins critical to this process is Beclin-1, the mammalian orthologue of the yeast autophagy protein Atg6. Beclin-1 induces autophagy by interacting with several cofactors and Vps-34, a class III phosphatidylinositol-3-phosphate kinase (PI3kIII/Vps34), to form a complex that promotes autophagosome formation in the early stages of autophagy [14]. Unlike Beclin-1, another member of the ATG protein family, microtubule-associated protein 1 light chain 3 (LC3) localize to different autophagic membranes and is essential for final autophagosome formation. LC3, synthesized as a pro-protein is rapidly converted to LC3-I by Atg4 and further transformed by a series of conjugating and activating Atg proteins to LC3-II, the autophagosomal membrane bound form considered as a marker of autophagy activation.

Selenium is central component at the catalytic sites of various selenium-dependent enzymes including Glutathione peroxidase (GPx). Selenium has been demonstrated to reduce ROS production [15, 16], protect cells against glutamate toxicity [17], oxidative stress [18] and inflammatory cytokines [19, 20]. Animal experiments have showed that selenium supplementation reduces ROS production, ameliorates cisplatin induced neurotoxicity and ischemia-induced brain damage [21, 22]. However, the mechanisms by which selenium exerts its protective effect against glutamate insult in neuronal cells remains ambiguous. In this study, glutamate exposed HT22 cells were used as an in vitro model to examine signaling pathway through which, sodium selenite could potentially moderate glutamate-induced toxicity in neuronal cells. Cellular viability, ultrastructural changes, ROS production, and biomarkers for autophagy and mitochondrial fission were measured respectively. Our results showed that sodium selenite blocked glutamate provoked neuronal death by restoring mitochondrial function, reducing ROS production, inhibiting caspase activation, and impairment in autophagy.

## Results

### Glutamate decreased and selenite increased cell viability

**Fig. 1:**
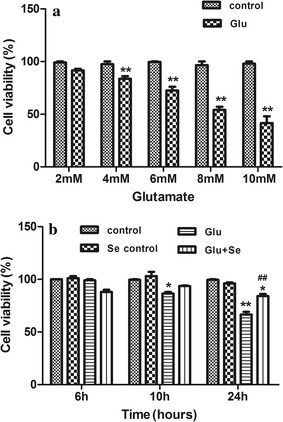
Cell viabilities in three experimental groups assessed by MTT assay. **a** cell viability assays were performed at 24 h of incubation with various concentrations of glutamate. The viabilities of the cells were reduced to 94.2 ± 0.6, 85.2 ± 1.4, 72.0 ± 1.6, 54.2 ± 1.6, and 42.5 ± 2.4% with the glutamate at 2, 4, 6, 8, and 10 mM concentrations. N = 10 in each group. **p < 0.01 versus control by Student’s *t* test. **b** Cell viability after 6, 10 and 24 h of glutamate (6 mM) incubation with or without concurrent addition of selenium (100 nM). After 10 h of incubation, cell viabilities were reduced to 85.8 ± 2.5% by glutamate and the reduction was moderately improved by selenite (92.2 ± 1.3%). F(3, 23) = 13.6 and p < 0.001 by ANOVA. After 24 h of culture, glutamate further reduced the cell viability to 68.9 ± 3.9%, while selenite rescued the viability to 83.6 ± 1.5%. F(3, 23) = 18.9. *p < 0.05, **p < 0.01 versus control and ^##^ *p* < 0.01 versus glutamate by ANOVA with post hoc Tukey’s test. *Glu* glutamate, *Se* sodium selenite, *Glu* *+* *Se* = glutamate + sodium selenite. N = 6 in each group

The cell viabilities in vehicle and various concentrations of glutamate treated cells were assessed using MTT assay kit after 24 h of incubation (Fig. 1a). The results showed that incubation with 2 mM of glutamate reduced the cell viability from 98.8 ± 1.2% in control to 94.2 ± 0.6% (p > 0.05), while 4 mM of glutamate reduced it to 85.2 ± 1.4% (p < 0.01 compared with control). The cell viability was further decreased with the increased concentrations of glutamate. Therefore, the cell viabilities were 72.0 ± 1.6% at 6 mM, 54.2 ± 1.6% at 8 mM, and 42.5 ± 2.4% at 10 mM of glutamate concentrations (p < 0.01 with all three concentrations compared with control). In next experiments, we incubated the cell with 6 mM glutamate and 100 nM of sodium selenite concurrently and measured cell viability after 6, 10 and 24 h (Fig. 1b). The results showed that the cell viabilities were not significantly altered in selenite, glutamate and glutamate plus selenite groups compared with vehicle control after 6 h of incubation. The cell viabilities were significantly decreased after 10 h of glutamate incubation to 85.8 ± 2.5% comparing to control of 100 ± 0.9% (p < 0.01). The viability was further decreased to 68.9 ± 3.9% after 24 h of glutamate incubation compared with control (p < 0.01). Compared with glutamate incubation alone, the HT22 cells co-treated with glutamate and selenite had a mild improved cell viability after 10 h (92.2 ± 1.3%, p > 0.05). However, the viability was significantly improved by selenite co-treatment at 24 h comparing to glutamate alone (83.6 ± 1.5% vs. 68.9 ± 3.9%, p < 0.01).

### Light and electron microscopic findings

**Fig. 2:**
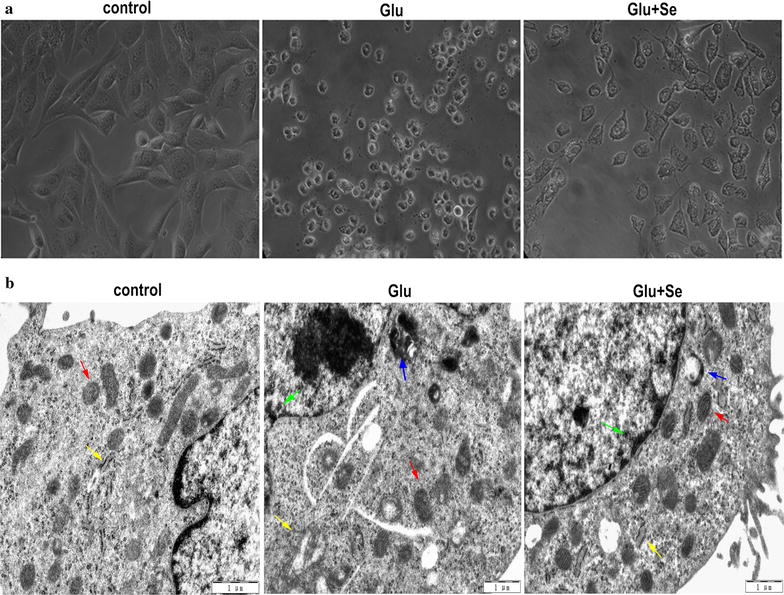
Cell morphology observed by light microscope (**a**) and electron microscope (**b**). **a** Glutamate exposure causes cell shrinkage and nuclear condensation. Selenite concurrent treatment reduced glutamate caused cell death. **b** Glutamate exposure 24 h caused mitochondrial lucency and swelling (*red arrow*), nuclear chromatin margination (*green arrow*), decreased presence of subcellular organelles, distention of rough endoplasmic reticulum (*yellow arrow*), and increased presence of lysosome (*blue arrow*). Selenite concurrent treatment reduced glutamate-caused alterations in the mitochdrion, endoplasmic reticulum and lysosome

Consistent to the results obtained from viability assay, cell images captured by inverted light microscope demonstrated normal cell morphology in non-glutamate treated control HT22 cells. Glutamate exposure resulted in cell shrinkage and nuclear condensation, suggesting cell death (Fig. 2a). Selenite concurrent treatment ameliorated glutamate-caused cellular morphological changes. Electron microscopic study showed that abundant endoplasmic reticulum (yellow arrow) and mitochondria (red arrow), mostly elongated, in the cytosol of the control cells. Comparing to the control, mitochondria in glutamate-treated cells became lucent and swelling (red arrow). In addition, distended rough endoplasmic reticulum, abundant presence of lysosomes (blue arrow), autophagosomes, and nuclear chromatin margination (green arrow) were observed. Selenite concurrent treatment ameliorated glutamate caused alterations in the mitochondrion, endoplasmic reticulum and lysosome (Fig. 2b).

### Selenium decreased glutamate-induced superoxide production

**Fig. 3:**
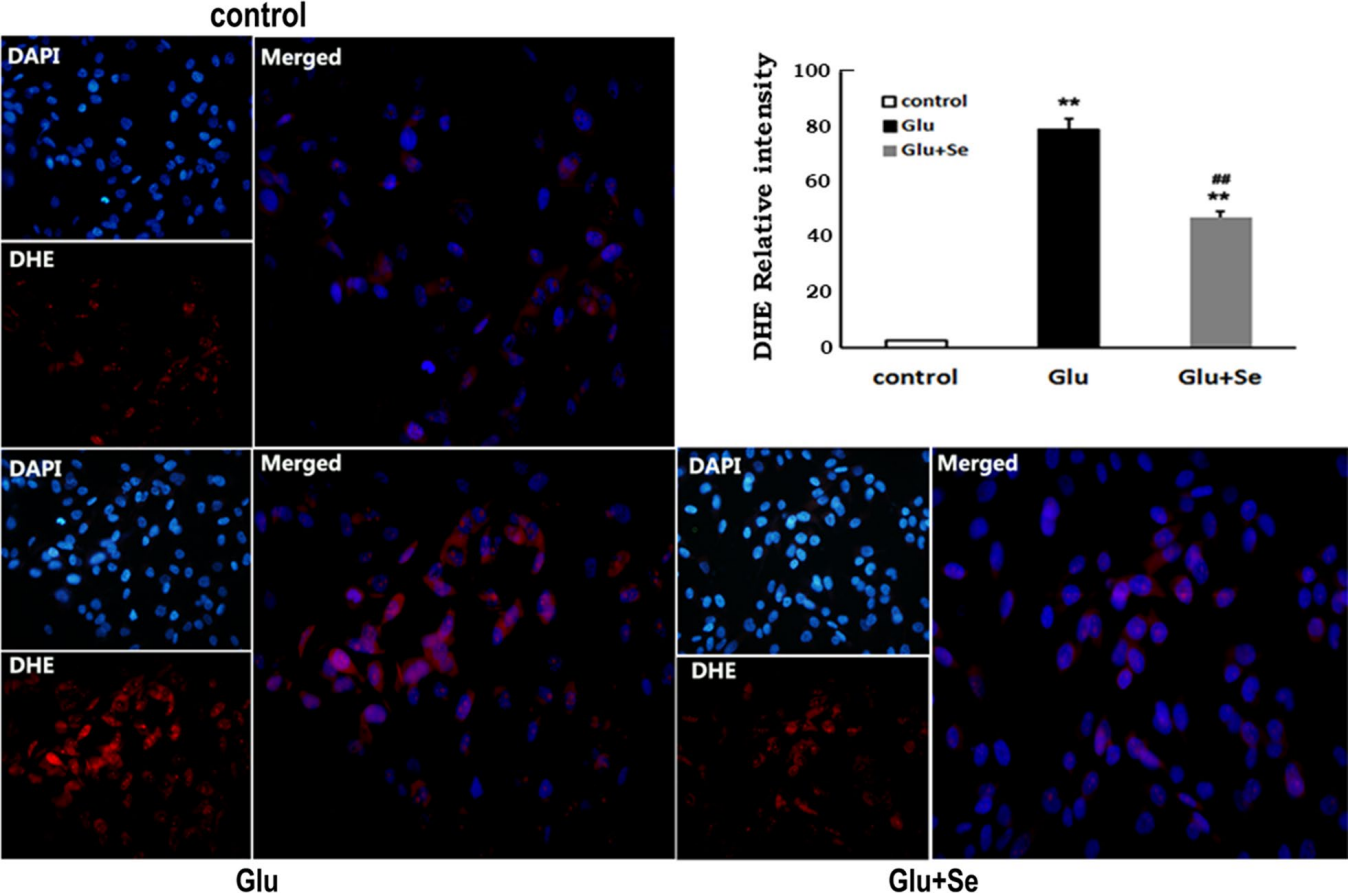
Superoxide accumulation measured by dihydroethidium (DHE) fluorescence. Accumulation of superoxide in the cells was measures by incubation in DHE (*red color*) after 24 h following glutamate and/or selenium treatments. Cells were counterstained with DAPI to label the nuclei (*blue color*). Relative immunointensity increased from 6.0 ± 0.8 in control to 81.0 ± 2.1 after glutamate exposure. Concurrent treatment of the cells with glutamate and selenite reduce the DHE immunointensity to 47.0 ± 1.2. F(2, 23) = 633.5. **p < 0.01 versus control and ^##^ *p* < 0.01 versus glutamate by ANOVA with post hoc Tukey’s test. N = 10 in each group

Superoxide productions were measured by DHE fluorescent probe following glutamate and/or selenite incubation. As shown in Fig. 3, faint DHE immunofluorescence was detected in normal HT22 control cells, representing the basal amount of superoxide (value of 6.0 ± 0.8) produced during normal cellular metabolic activity. Glutamate exposure resulted in a surge of DHE signal (value of 81.0 ± 2.1), suggesting significantly increased superoxide production (p < 0.01). Treatment with selenium in glutamate-incubated cells reduced the DHE fluorescent intensity to 47.0 ± 1.2, a 42% reduction comparing to that of non-selenite treated, glutamate exposed cultures (p < 0.01).

### Glutamate activated and selenium suppressed mitochondria-initiated cell death pathway

**Fig. 4:**
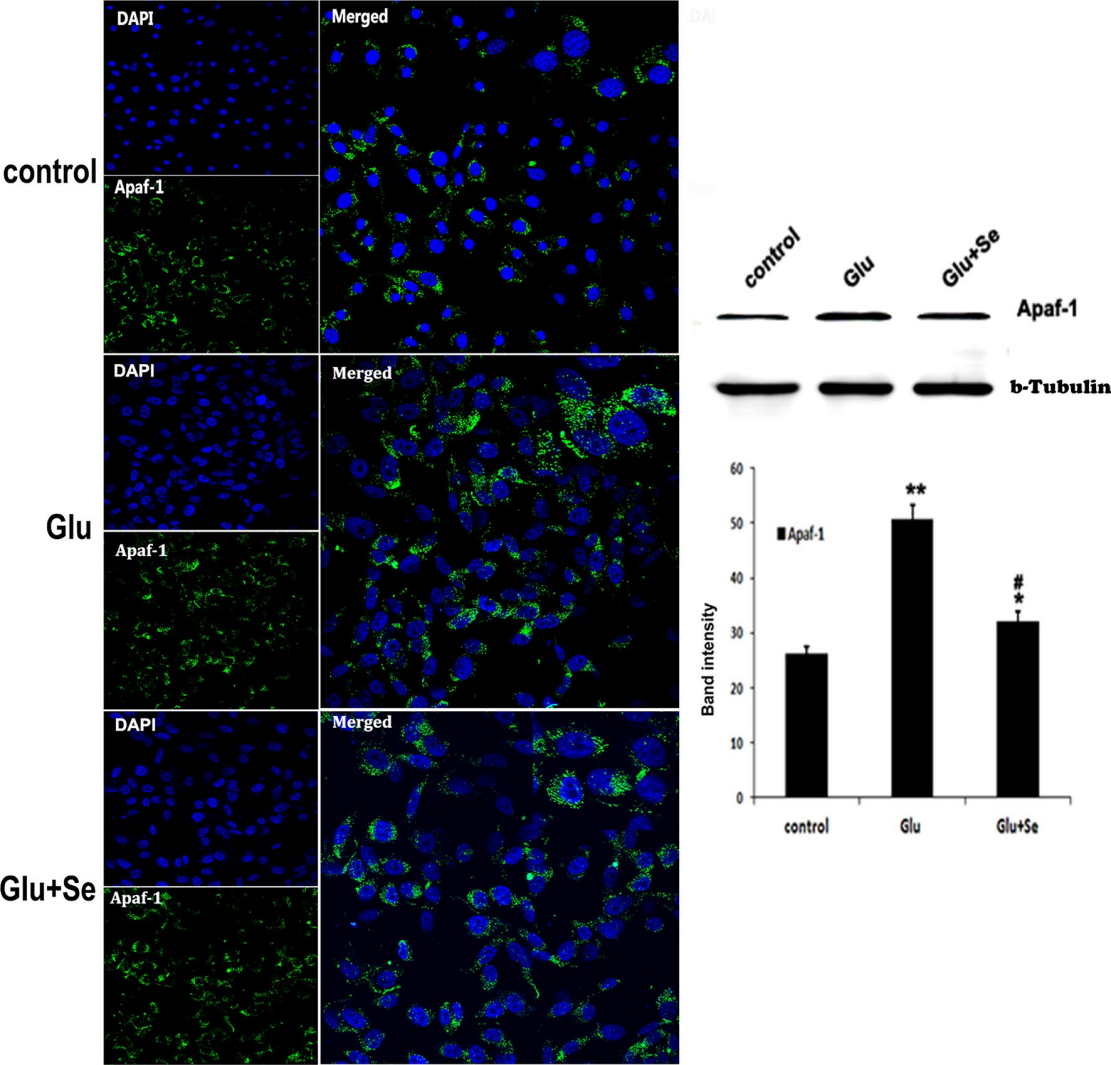
Immunocytochemistry and protein blotting of Apaf-1. Apaf-1 (*green color*) was detected using antibody after 24 h of glutamate or glutamate plus selenite treatments. DAPI (*blue color*) was used to label the nuclei. Western blot was performed using cytosolic fraction. Relative band intensity was measured and presented in the *bar graph*. Β-tubulin served as internal protein loading control. Glutamate incubation increased the Apaf-1 protein band intensity to 51.8 ± 1.4 and concurrent treatment with selenite reduced the value to 32.7 ± 1.4. F(2, 29) = 97.1. *p < 0.05 and **p < 0.01 versus control; ^#^ *p* < 0.05 versus Glutamate by ANOVA with post hoc Tukey’s test. N = 10 in each group

Since Apaf-1, caspase-9, and caspase-3 play key roles in mediating mitochondria-initiated cell death pathway, we performed immunocytochemistry using antibodies against Apaf-1, cleaved caspase-9, and cleaved caspase-3. The results were further verified with Western blots using antibodies against Apaf-1, total and cleaved caspase-9, and total and cleaved caspase-3. The results showed that there were very few faintly stained Apaf-1 positive neurons in the normal control cells. Glutamate exposure for 24 h significantly increased the immunoreactivity of Apaf-1 in the cytoplasm as shown in Fig. 4. Concurrent treatment with selenite reduced the immunoreactivity of Apaf-1 in glutamate-exposed neurons. Western blot using cytosolic fraction further support the findings showing that Apaf-1 protein band intensity in the cytosolic fraction were significantly increased from 25.3 ± 1.4% in control to 51.8 ± 1.4 in glutamate treated cells (p < 0.01). Selenite treatment significantly reduced the Apaf-1 protein band intensity to 32.7 ± 1.4 (p < 0.05 vs. glutamate treatment).

**Fig. 5:**
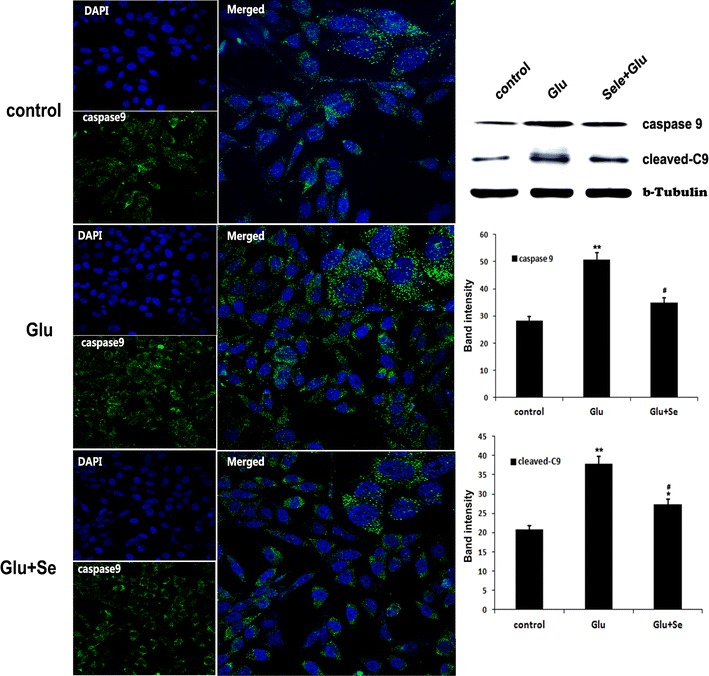
Immunocytochemistry and protein blotting of caspase-9. Immunocytochemistry was performed 24 h after treatments using antibody against cleaved caspase-9 (*green color*) and counterstained with DAPI (*blue color*). Western blots were performed using antibodies against total and cleaved caspase-9 in the cytosolic fraction. Beta-tubulin served as an internal control. Relative band intensities were measured and presented in the *bar graph*. Cleaved caspase-9 protein band intensity increased from 21.8 ± 1.8 in control to 38.4 ± 1.7 after glutamate incubation and reduced to 27.1 ± 1.1 by selenite treatment. F(2, 29) = 102.4. *p < 0.05, **p < 0.01 versus control; ^#^ *p* < 0.05 versus Glutamate by ANOVA with post hoc Tukey’s test. N = 10 in each group

Similarly, there were very few faintly stained caspase-9 positive cells in control cultures. After glutamate treatment for 24 h, the number of caspase-9 positively stained neurons increased and concurrent treated of selenium decreased immunointensity of caspase-9 (Fig. 5). The results were further confirmed by Western blot. Consistently, glutamate caused significant elevation of cleaved caspase-9 (38.4 ± 1.7) compared to control (21.8 ± 1.8). Treatment with selenite significantly ablated the glutamate-induced increase (27.1 ± 1.1).

**Fig. 6:**
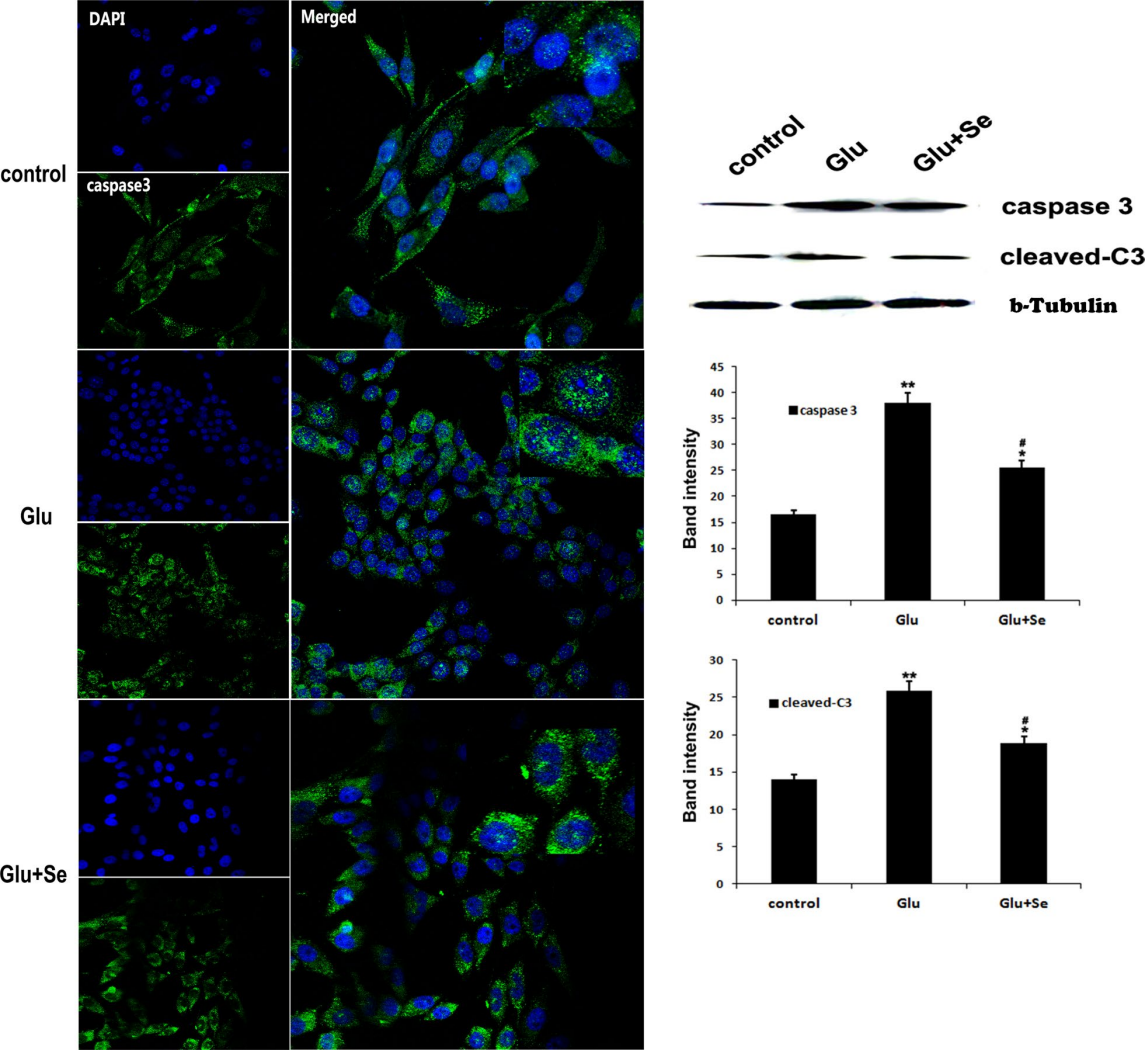
Immunocytochemistry and protein blotting of caspase-3. Immunocytochemistry was performed 24 h after treatments using antibody against cleaved caspase-3 (*green color*) and counterstained with DAPI (*blue color*). Western blots were performed using antibodies against cleaved caspase-3 in the nuclear fraction. Beta-tubulin served as an internal control. Relative band intensities were measured and presented in the *bar graph*. Cleaved caspase-3 protein band intensity increased from 13.6 ± 0.8 in control to 25.5 ± 1.5 after glutamate incubation and concurrent treatment of selenite reduced it to 18.7 ± 1.2. F(2, 29) = 67.5. *p < 0.05 and **p < 0.01 versus control; ^#^ *p* < 0.05 versus Glutamate by ANOVA with post hoc Tukey’s test. N = 10 in each group

There was faint punctuated caspase-3 positive staining localized in the cytoplasm of non-glutamate-incubated control cells. After glutamate incubation for 24 h, caspase-3 immunoreactivity significantly enhanced in the cytosol and nuclear translocation was noted. Concurrent treatment with selenite prevented caspase-3 nuclear translocation but did not seem to reduce the immunoreactivity in the cytosol and prevented caspase-3 nuclear translocation (Fig. 6). Western blot using nuclear fraction revealed that cleaved caspase-3 increased significantly in glutamate-incubated cells (25.5 ± 1.5) compared with control (13.6 ± 0.8). Such increases were ameliorated by selenite treatment (18.7 ± 1.2).

### Selenium reduces glutamate-induced mitochondrial fission

**Fig. 7:**
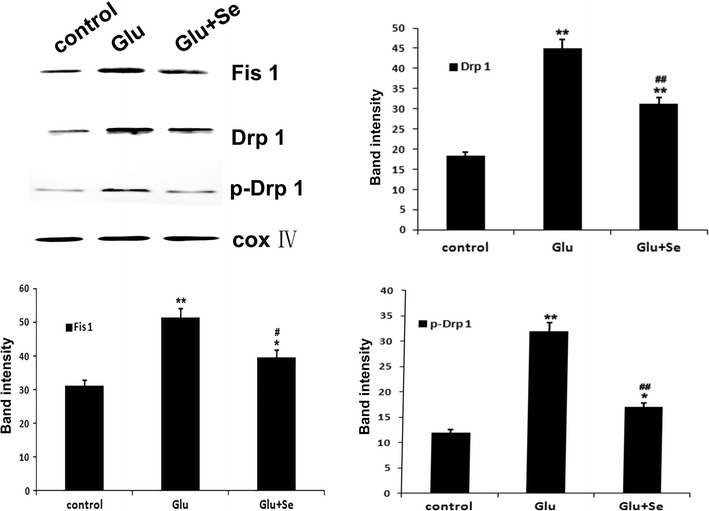
Western blotting of mitochondrial fission proteins. Fission proteins Fis-1, total Drp-1 and phospho-Drp1 (p-Drp1) were semi-quantified in the mitochondrial fractions using specific antibodies. COX IV served as an internal control. Band relative densities were measured and presented in the *bar graphs*. Fis-1 protein band intensity increased from 31.4 ± 1.3 in control to 51.1 ± 1.4 after glutamate incubation and reduced back to 40.5 ± 1.3 by selenite. F(2, 29) = 24.2. *p < 0.05 and **p < 0.01 versus control; ^#^ *p* < 0.05 and ^##^ *p* < 0.01 versus Glutamate by ANOVA host hoc Tukey’s test. Similarly, p-Drp-1 ban intensity increased from 12.3 ± 1.1 in control to 32.3 ± 1.4 in glutamate and declined to 16.6 ± 1.0 in selenite and glutamate co-treated cells. F(2, 29) = 54.7. *p < 0.05 and **p < 0.01 versus control; ^#^ *p* < 0.05 and ^##^ *p* < 0.01 versus Glutamate by ANOVA with post hoc Tukey’s test. N = 10 in each group

Mitochondrial fission markers Fis1, Drp1, and p-Drp1 were detected in the mitochondrial fraction of the cell lysate using Western blotting. As shown in Fig. 7, Protein band intensity of Fis1 increased significantly in glutamate treated cells (51.1 ± 1.4) comparing to control cells (31.4 ± 1.3). Concurrent treatment of glutamate incubated cells with selenite significantly reduced the level of Fis1 to 40.5 ± 1.3 (p < 0.01). Similarly, glutamate increased the p-Drp1 protein relative band intensity from 12.3 ± 1.1 in control to 32.3 ± 1.4 in glutamate incubated cells. Treatment with selenite reduced the p-Drp1 intensity to 16.6 ± 1.0 (p < 0.01). This is further supported by the following imaging study.

### Selenium suppressed glutamate-activated autophagy

**Fig. 8:**
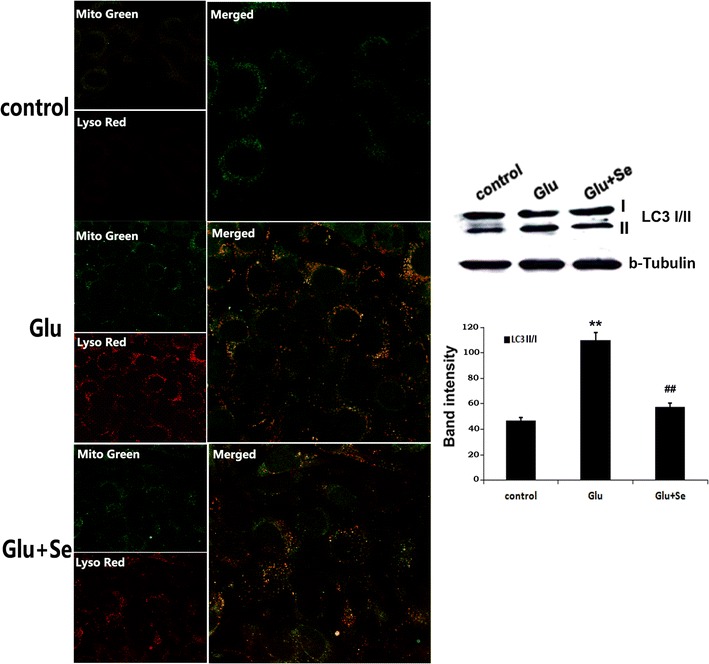
Co-localization of MitoTracker Green and LysoTracker Red. The cells were incubated for 30 min with MitoTracker Green (*green color*) and LysoTracker Red (*red color*) to visualize the colocalization of mitochondria and lysosomes. Colocalization of the two dyes turns the color into orange. Western blot detected LC3-I and II in the mitochondrial fraction. Relative LC3-II band intensities were measured and presented in the *bar graph*. LC3-II protein band intensity increased from 46.8 ± 1.7 in control to 105.4 ± 4.2 in glutamate and reduced to 57.4 ± 1.8 in selenite treated groups. F(2, 29) = 124.9. *p < 0.05 and **p < 0.01 versus control; ^##^ *p* < 0.01 versus glutamate by ANOVA with post hoc Tukey’s test. N = 10 in each group

The last step of autophagy is the fusion of autophagosome with lysosome. Therefore, colocalization of mitochondrial marker MitoTracker Green and lysosomal marker LysoTracker Red could be used as an indication of mitochondrial autophagy. As shown in Fig. 8, in control cells, punctate green staining was observed, representing normal mitochondria detected by the probe. There were almost no lysosomes were detected. After glutamate treatment for 24 h, both MitoTracker Green and LysoTracker Red staining increased significantly in the cytosol, suggesting increased numbers of mitochondria and lysosomes. Treatment with selenite reduced the intensity of LysoTracker labeling.

Another marker of autophagy is the conversion of LC3-I to LC3-II. LC-3 levels were measure in the cytosolic fraction of the cells. It was shown that the levels of LC3-II band intensities significantly increased from 46.8 ± 1.7 in control to 105.4 ± 4.2 in glutamate treated cells (p < 0.01) and selenite reverted the glutamate-induced elevation back to 57.4 ± 1.8 (p < 0.01).

## Discussion

Neuronal cell injury and death are major pathological features of many CNS disorders such as Alzheimer’s disease, Parkinson’s disease, Huntington’s disease, and stroke. These disorders provoke alterations in cell function and survival in distinct regions of the brain. For example, stroke triggers a significant neuronal death and several theories, including excitotoxicity, production of oxygen free radicals, nitric oxide level, and mitochondrial injury have been proposed to explain the cause of damage to neurons during stroke [23, 24]. Ischemic stroke occurs as a result of focal cerebral ischemia associated with permanent brain infarction. Ischemic stroke often leads to excessive glutamate release, acute excitotoxicity and nerve cell damage. In this study, HT22 cells exposed to glutamate was used as in vitro model of stroke to explore the effects of selenium on glutamate excitotoxicity and to assess the underlying mechanisms that lead to attenuation of glutamate-induced toxicity in the cell. Our findings showed that selenium could attenuate cell apoptosis, diminish superoxide production, and decrease expression of Apaf-1, caspase 9, and caspase 3. In addition, selenium decreased the level of the LC-3II, a proxy for autophagosome and protected against mitochondrial impairment induced by glutamate. Our results further indicate that glutamate-induced cell death appears to be a results caused by a mechanism that involves structural changes typical of apoptosis, including swelling of the mitochondria, chromatin condensation, and appearance of lysosomes.

Selenium has traditionally been used as a supplement for several decades in the prevention and treatment of human diseases including Keshan cardiomyopathy [25], Kashin-Beck osteoarthropathy [26, 27], osteopenia [28] and certain forms of cancers [29–31], and has been shown to modulate the functions of a variety of intracellular proteins [32–34]. Recent research has focused on the neuroprotective potential of selenium as a candidate for therapeutic intervention in hypoxic injuries and ischemia-induced neuronal damage [35–39]. The neuroprotective effect of selenium may be dependent on its antioxidant properties. Selenium in the form of selenoprotein is present in the active site of glutathione peroxidase and regulates the activity of the enzyme. Glutathione peroxidase participates in the complex antioxidant defense signaling network that is essential for the detoxification of cellular stresses induced by free radicals. ROS are chemically reactive molecules that contain oxygen such as superoxide, singlet oxygen, and hydrogen peroxide (H_2_O_2_). They are produced as natural byproducts of normal metabolism and play beneficial roles in cell signaling [40]. However, excessive ROS can be detrimental to cells as a result of induction of oxidative stress and cellular damage [41]. Earlier studies describing the chronic effect of glutamate exposure in cellular toxicity point to the crucial role of oxidative stress-induced ROS as a major stimulus of glutamate-induced cell death [17, 42–45]. It has been reported that excessive concentrations of extracellular glutamate antagonize the glutamate/cystine-antiporter and inhibit cystine uptake. This inhibitory effect leads to accumulation of ROS, oxidative stress, and mitochondrial hyperactivation [46]. Previous studies have shown that selenium attenuates the level of ROS following various in vitro injury models [16, 47–49]. Our results are in agreement with these studies. We observed high production of cellular superoxide radical with glutamate treatment that was considerably reduced upon co-treatment with selenium. This suggest that selenium exerts its influence partly by enhancing the activity of antioxidant systems in cells under stress.

Apoptosis, also known as programmed cell death, is a tightly regulated cell suicide process necessary for growth, development, and aging in multicellular organisms. Two well defined apoptotic pathways, intrinsic and extrinsic pathways, have been identified. Both pathways can be are activated by a variety of physiological and pathological conditions or stimuli and converge on a common execution point driven by caspases. Caspases are a family of cysteine-aspartic proteases endoproteases. Caspases are generally divided into two subtypes, initiator and effector caspases. Initiator caspases (caspases-8, -9 and -10) contain large prodomains and act as regulators of the caspase proteolytic cascade. Effector caspases (caspases-3, -6 and -7) are activated by initiator caspases, through proteolytic cleavage, and function in disassembly of cells into apoptotic bodies [50]. Glutamate induces cell death through processes that involve both necrosis and apoptosis [44, 51] although, apoptosis appears to be the major driver of cell fate at late time points. Apoptotic process culminate in alterations in the integrity of the inner mitochondrial membrane that results in the formation of the MPTP [52], failure of the mitochondrial membrane potential and release of pro-apoptogenic proteins into the cytosol. Among the proteins released from the mitochondria is Cytochrome c (Cyt c), a major component of the electron transport chain. Cyt c binds and activates Apaf-1, recruits procaspase-9 and dATP, and thus facilitates assembly of the apoptosome. Formation of the multi-protein apoptosome complex results in the activation of effector caspases 3, 6, and 7, which in turn translocates to the nucleus, and initiates cleavage of DNA and subsequent degradation of nucleosomal proteins [53]. Our findings show that the magnitude of Apaf-1, increased significantly in HT-22 cells under glutamate tension, while selenium antagonized the glutamate-induced increase in Apaf-1. Furthermore, glutamate-dependent increase in Apaf-1 was accompanied by concomitant increases in cleaved caspase 9 and cleaved caspase 3. Our data indicate that selenium also reduced the stimulation of caspase 9 and caspase-3-like protease activity that occurs in glutamate exposed cells. These selenium-dependent influences on effector caspase have been demonstrated previously in other cellular and in vivo animal models [15, 16, 54]. Apaf-1 is one of the well-studied physiological regulator of events of mitochondria-dependent apoptosis in most death pathways. Typically, it exists in an auto-inhibited form in the cytosol until it binds to Cyt c and promotes assembly of the apoptosome [55]. Our present findings suggest that inhibition of Apaf-1 activity may play a central role as a potential mechanism by which selenium protects HT22 cells from glutamate excitotoxicity.

The structure of mitochondria is mainly regulated by cycles of fusion and fission. During apoptosis, mitochondria undergo fission, chromatin condensation, and becomes highly fragmented [56]. The mammalian Drp1 is a dynamin-related cytoplasmic GTPase that mediates mitochondrial fission. Drp1 is a highly regulated protein and undergoes a number of steps including translocation from cytoplasm to the mitochondrial outer membrane for fission to occur [57, 58]. Our results showed that p-Drp1, as well as Fis-1, increased significantly after glutamate exposure, suggesting that glutamate triggered mitochondrial fission. Selenium treatment hampered the increases of p-Drp1 and Fis-1, indicating its ability of preventing mitochondrial fission. It is widely accepted that fragmentation associated with mitochondrial fission is a key activator of mitochondrial autophagy. During autophagy, components of damaged mitochondria are encapsulated in autophagosomes. Progression of autophagy is dependent on the recruitment of LC3-II, a c-terminal lipidated form of cytosolic LC3-I as a structural component of the autophagosomal membrane. Thereafter, LC3-II tagged autophagosomes are delivered to the lysosome/vacuole for degradation by lysosomal hydrolases. Our results revealed that glutamate increased the presence of lysosome in the cytoplasm and colocalization with the mitochondrial marker, supporting the prediction that glutamate induces mitochondrial autophagy. The presence of autophagy was supported by quantifying the relationship between LC3-I and LC3-II. Glutamate exposure led to an increase in autophagy as reflected in the conversion of LC3-I to LC3-II using Western blotting. Selenium on the other hand, significantly reduced the intensity of LysoTracker labeling and blocked the alteration of LC3-I to LC3-II, and thus, protected against glutamate-dependent cell death. Increased levels of Beclin 1 and upregulation of LC3-II relative to LC3-I as markers of autophagy in cells exposed to glutamate have been reported in our earlier studies [17]. Although the mechanisms by which selenium altered the ratios of LC3-I and LC3-II in the presence of glutamate is uncertain, we posit that selenium could modulate this process potentially by blocking the upstream conjugation of phosphatidylethanolamine to LC3-I, or enable interconversion of LC3-II back to LC3-I. The actual mechanism(s) remains to be elucidated.

## Conclusions

Our results indicate that glutamate exposure causes increase of superoxide radical, activation of mitochondria-initiated cell death pathway, tilting mitochondrial dynamic balance towards fission and triggers mitochondrial autophagy. Concurrent treatment with sodium selenite rescues glutamate-induced cell death by reducing superoxide production, inhibiting intrinsic cell death pathway, blocking mitochondrial fission and suppressing mitochondrial autophagy. It is concluded that the protective effects of selenium against glutamate cytotoxicity is associated with preserving mitochondrial integrity and dynamic balance.

## Methods

### Cell culture and materials

Murine hippocampal neuronal HT22 cells (originally purchased from ATCC) were cultured in Dulbecco’s Modified Eagle Medium (DMEM)/F12 (Invitrogen, Carlsbad, CA), supplemented with 10% fetal bovine serum (FBS, HyClone), 100 nM penicillin/streptomycin, at 37 °C in 5% CO_2_ and 90% relative humidity. The culture medium was renewed every 2 days. Sodium glutamate, and sodium selenite (Sigma-Aldrich, St Lois, MO), were reconstituted in water and diluted to appropriate concentrations in cell culture medium. Antibodies directed against caspase-3, caspase-9, apoptosis protease-activating factor-1 (Apaf-1), cleaved caspase-3, cleaved caspase-9 were purchased from Cell Signaling Technology, Danvers, MA. Anti LC3A/B, Fis1, Drp1, p-Drp1, and β-actin were purchased from Abcam, Cambridge, MA.

### Cell viability assay

Cell viability was assessed with MTT cell proliferation assay kit (Trevigen Inc., Gaithersburg, MD) according to manufacturer’s instructions. In brief, HT22 cells were plated into 96-well plates (5000/well, Corning Inc., New York, NY). After seeding 24 h the cultures exposed to various concentrations of glutamate (2, 4, 6, 8, and 10 mM). After 24 h of incubation, the results were read in the SpectraMax microplate reader (Molecular Devices, Sunnyvale, CA). Glutamate of 6 mM was selected for the subsequent experiments.

### Selenium treatment

Sodium selenite (100 nM) was added to the media concurrently with the glutamate. The selection of selenium concentration was based on results of previous publications [17, 23], which showed that 100–200 nM selenium provided the adequate neuroprotection in vitro, while 500 nM was toxic to the cells.

### Electron microscopy

Log-phase cultures were collected, centrifuged and the pellets were fixed with 2% glutaraldehyde solution for 2 h at 4 °C. The samples were incubated in osmic acid for 2 h and 0.1 M dimethyl sodium arsenate buffer for 15 min for 2 times at 4 °C. Thereafter, the pellets were dehydrated in ascending ethanol, rinsed in propylene oxide, and incubated in propylene oxide and resin before embedding resin. The pellets were sliced and imaged on a transmission electron microscope.

### Determination of superoxide radical

Superoxide anion production was measured by dihydroethidine (DHE) in glutamate only or selenium-co-treated HT22 cells exposed to glutamate (6 mM). Briefly, cells (5000 per well) were seeded on glass slides. After 24 h treatment period, the slides were incubated with the DHE (50 μM) for 45 min at 37 °C, washed with PBS, and fixed in 4% paraformaldehyde. Images were captured under 400× magnification using a fluorescent microscope (Olympus CHC-212) and fluorescent intensity was measured by IPP6.0 software.

### Western blotting

For immunoblotting, treated cells were lysed in RIPA buffer containing protease (Roche Diagnostics Corp., Indianapolis, IN) and phosphatase (ThermoFisher Scientific, Waltham, MA) inhibitor cocktails.

The cells were lysed and subcellular fractions of the cytosol, mitochondrion, and nucleus were isolated through various peed of centrifugations [17]. Twenty micrograms of proteins in the total cell lysates were separated on 4–12% NuPAGE SDS-PAGE gels (Invitrogen), transferred to nitrocellulose membrane, and probed with the following antibodies:anti-Apaf-1 (1:500) and anti-caspase-9 (1:1000) for the cytosolic fraction; anti-caspase-3 (1:500) for the nuclear fraction; and anti-LC3A/B (1:1000), Fis 1 (1:2000), Drp1 (1:2000), p-Drp1 (1:1000) for the mitochondrial fraction. Anti β-actin (1:1000) was used as internal control for protein loading. After washing, the membranes were incubated in HRP-conjugated secondary anti IgG (1:5000) for 3 h at room temperature. Antibody binding was detected by enhanced chemiluminescence using Gel imaging analyzer (BIO-Rad Inc., Hercules, CA) and band intensity was measured using Image-J2x software.

### Immunofluorescence

Cells were seeded for 24 h on glass slides and treated with glutamate or glutamate and selenium for additional 24 h in a humidified incubator at 37 °C and 5% CO_2_. The slides were washed with PBS, fixed with 4% paraformaldehyde for 15 min and permeabilized with 1% TritonX-100 for 30 min. The nonspecific binding sites were blocked with 10% goat serum and incubated overnight at 4 °C with antibodies against Apaf-1 (1:100), cleaved caspase-3 (1:100), and cleaved caspase-9 (1:100). After washing 3 times in PBS the slides were incubated secondary antibody (1:200) for 1 h at 4 °C and DAPI for 5 min in a dark environment. Images were captured in each well at a magnification of 400× and fluorescence intensities were measured as describe previously.

### Detection of autophagy

Autophagy was detected by immunofluorescent double labeling with LysoTracker Red and MitoTracker Green. Cells were seeded in 12-well plate and treated with glutamate and selenium as previously described. Following treatment, LysoTracker Red (75 nM), was added to the culture medium and the cells were returned to the incubator for 45 min. MitoTracker Green (100 nM) was then added into culture medium an incubation continued for additional 45 min in a humidified incubator at 37 °C and 5% CO_2_. The slides were washed with PBS and fixed with 4% paraformaldehyde. Images were captured under the magnification of 400× using a fluorescent microscope (Olympus CHC-212) and fluorescent intensity were measured by IPP6.0 software.

### Statistics

All experiments were repeated at least 2 times and carried out in triplicate. Statistical analysis was performed with IBM SPSS Statistics 19.0. All values were expressed as mean ± SEM. Data in Fig. 1a were analyzed by Student’s *t* test. Data in Figs. 1b and 3, 4, 5, 6, 7 and 8 were analyzed by one-way ANOVA and followed by Tukey’s test to detect differences between groups. Statistical significance was set at p < 0.05.

## Abbreviations

AD: Alzheimer’s disease
ANOVA: analysis of variance
Apaf-1: apoptosis protease-activating factor-1
Cyt c: cytochrome c (Cyt c)
DMEM: Dulbecco’s Modified Eagle Medium
DAPI: 4′,6-diamidino-2-phenylindole
DHE: dihydroethidium
Drp1: dynamin-like protein 1
FBS: fetal bovine serum
Fis1: fission 1 protein
GPx: glutathione peroxidase
GSH: glutathione
HRP: horseradish peroxidase
H_2_O_2_: hydrogen peroxide
LC3: microtubule-associated protein 1 light chain 3
Mfn: mitofusin
MPTP: mitochondrial permeability transition pore
Opa1: optic atrophy 1
PD: Parkinson’s disease
RIPA buffer: radioimmunoprecipitation assay buffer
ROS: reactive oxygen species
SEM: standard error of the mean
